# Transcriptomic profiling of genes in matured dimorphic seeds of euhalophyte *Suaeda salsa*

**DOI:** 10.1186/s12864-017-4104-9

**Published:** 2017-09-13

**Authors:** Yange Xu, Yuanqin Zhao, Huimin Duan, Na Sui, Fang Yuan, Jie Song

**Affiliations:** Shandong Provincial Key Laboratory of Plant Stress, College of life science, Shandong Normal University, Jinan, Shandong 250014 People’s Republic of China

**Keywords:** Dimorphic seed, Seed germination, Transcriptome, Plant hormone, *Suaeda salsa*

## Abstract

**Background:**

*Suaeda salsa* (*S. salsa*) is a euhalophyte with high economic value. *S. salsa* can produce dimorphic seeds. Brown seeds are more salt tolerant, can germinate quickly and maintain the fitness of the species under high saline conditions. Black seeds are less salt tolerant, may become part of the seed bank and germinate when soil salinity is reduced. Previous reports have mainly focused on the ecophysiological traits of seed germination and production under saline conditions in this species. However, there is no information available on the molecular characteristics of *S. salsa* dimorphic seeds.

**Results:**

In the present study, a total of 5825 differentially expressed genes were obtained; and 4648 differentially expressed genes were annotated based on a sequence similarity search, utilizing five public databases by transcriptome analysis. The different expression of these genes may be associated with embryo development, fatty acid, osmotic regulation substances and plant hormones in brown and black seeds. Compared to black seeds, most genes may relate to embryo development, and various genes that encode fatty acid desaturase and are involved in osmotic regulation substance synthesis or transport are upregulated in brown seeds. A large number of differentially expressed genes related to plant hormones were found in brown and black seeds, and their possible roles in regulating seed dormancy/germination were discussed.

**Conclusions:**

Upregulated genes involved in seed development and osmotic regulation substance accumulation may relate to bigger seed size and rapid seed germination in brown seeds, compared to black seeds. Differentially expressed genes of hormones may relate to seed dormancy/germination and the development of brown and black seeds. The transcriptome dataset will serve as a valuable resource to further understand gene expression and functional genomics in *S. salsa* dimorphic seeds.

**Electronic supplementary material:**

The online version of this article (10.1186/s12864-017-4104-9) contains supplementary material, which is available to authorized users.

## Background

At present, irreversible soil salinization has been a global issue, which not only affects the sustainable development of agriculture, but also becomes a potential threat of desertification [1]. Salt stress is harmful to plant growth and yield in crops [2]. Therefore, it is essential to develop and utilize saline-alkali land by developing salt-tolerant crops (salt-tolerant germplasm), thereby improving saline soil [3].

Halophytes, which are defined as plants that naturally inhabit saline environments and benefit from salt in their growth cycle, could be the best choice to develop saline agriculture [4, 5]. *Suaeda salsa* L. (*S. salsa*) is an annual euhalophytic herb with succulent leaves, which can be utilized as food, medicine, forage and bioenergy, and plays an important role in the restoration of contaminated land [6]. The species could be a promising model to understand salt tolerance and develop saline agriculture in the future [6]. Seed dimorphism is a powerful germination strategy in unpredictable environments such as deserts and high salt soil areas [7]. *S. salsa* produces dimorphic seeds in one plant; that is, brown seeds with a soft, semi-transparent outer seed coat and black seeds with a hard, non-transparent outer seed coat [8, 9]. Brown seeds are bigger, more salt tolerant and can absorb water more quickly than black seeds [8]. It has been suggested that brown seeds could germinate in spring under high saline conditions, and black seeds may germinate in late spring and summer when soil-salt content is low due to rainfall [8, 9]. In addition, black seeds have a relatively long period of dormancy, and light is necessary for black seeds under high saline conditions during germination [10]. By producing dimorphic seeds, *S. salsa* selects the “hedge-betting” strategy to adapt to saline environments during germination [11].

Seed germination, which determines whether the plants can successfully establish their progeny, is critical in the life cycle of halophytes in saline environments [12]. Seed germination will be delayed or prohibited under salt stress for both halophytes and non-halophytes in general conditions [9, 13, 14]. Seed germination is a complicated process influenced by a large number of genes and environmental factors [15]. The characteristics of the seed itself perform a pivotal role in seed germination such as seed structures, developmental status of the embryo, compounds that are imported from the mother plant, and some factors that are produced by the embryo itself, including plant hormones [16]. In previous studies, a number of experiments on the germination of *S. salsa* have been performed to investigate the difference between black seeds and brown seeds. However, studies of molecular biology on the difference of dimorphic seeds themselves in the regulation of dormancy and germination have seldom been reported. Furthermore, there is no report on the functional genomics of *S. salsa* seeds, especially for differentially expressed genes (DEGs) in dimorphic seeds by transcriptome analysis.

In the present study, the transcriptome of brown and black seeds were analyzed by high-throughput Illumina RNA sequencing (RNA-seq). By comparing the transcriptome of black and brown seeds, large numbers of DEGs were identified. The results of the present study may provide further insight into this complex regulatory network, which may be related to seed development, dormancy and seed germination under saline conditions in *S. salsa*.

## Results

### Transcriptome sequencing and de novo assembly

A total of 194.87 million clean reads were obtained for assembly, and further analysis after trimming adapters were performed to filter out low quality reads. The reads from *S. salsa* were assembled into 11,824,856 contigs, with a mean length of 47.91 bp and a N50 length of 48 bp. By performing pair-end joining and clustering, contig libraries were assembled into 208,588 transcripts with a mean length of 1055.93 bp and N50 lengths of 1751 bp. Then, these transcripts were further clustered into unigenes. A total of 91,753 unigenes were obtained from *S. salsa* with a mean length of 701.53 bp and a N50 length of 1099 bp. The size distribution of these unigenes is shown in Additional file 1: Figure S1 and Table 1. All raw transcriptome data have been deposited in the National Center for Biotechnology Information (NCBI) SRA database, and the accession number can be found in the “Availability of supporting data”.

**Table 1:** The size distribution of contigs, transcripts and unigenes identified in the transcriptome of *S. salsa* dimorphic seeds

	Contigs	Transcripts	Unigenes
Total number	11,824,856	208,588	91,753
Total length	566,541,417	220,255,280	64,367,580
N50 length	48	1751	1099
Mean length	47.91	1055.93	701.53

### Selection and identification of DEGs

**Fig. 1:**
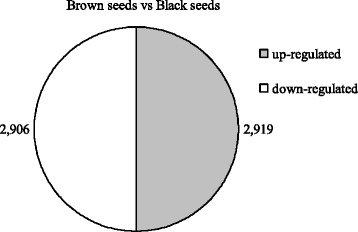
The number of DEGs of *S. salsa* dimorphic seeds

A total of 36,318 unigenes (39.58%) were identified with a significance threshold. A total of 5825 genes were found to be differentially expressed in black and brown seeds. Among them, 2919 genes were upregulated and 2906 genes were downregulated in brown seeds, compared to black seeds (Fig. 1).

### Functional annotation of DEGs

A total of 4648 DEGs were annotated against five public databases, and allowed the assignment of several functional annotations. DEG sequences were searched against the NCBI non-redundant (Nr) database [17] and Kyoto Encyclopedia of Genes and Genomes (KEGG) database [18], and 4641 and 1102 DEGs were annotated, respectively. In order to obtain more detailed protein annotations, the sequences were also blasted against the Swiss-Prot databases [19], and subsequently, 3410 DEGs were annotated. Furthermore, 1900 DEGs were annotated in the Clusters of Orthologous Groups (COG) databases [20], and 3659 DEGs were annotated into the Gene Ontology (GO) database [21].

### Functional classification by GO

**Fig. 2:**
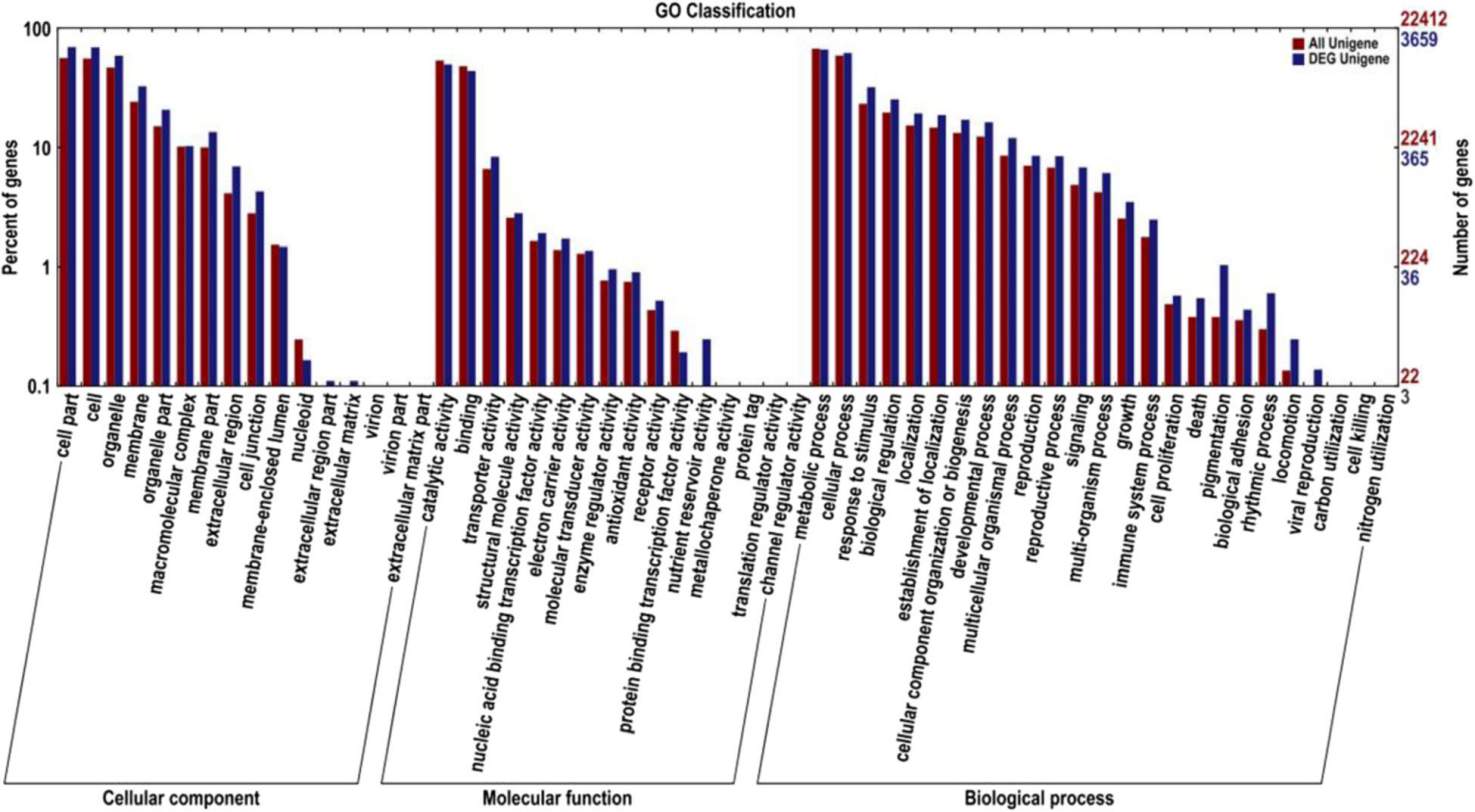
The gene ontology (GO) annotations of all annotated unigenes are shown. These results were summarized under the three main GO categories: biological process, cellular component and molecular function. The right Y-axis indicates the number of genes in each category. The left Y-axis indicates the percentage of a specific category of genes in the corresponding GO category

GO analysis provides a structured, precisely defined, dynamic, controlled vocabulary for describing the roles of genes and gene products in any organism with a biological process, molecular function and cellular component [21]. A total of 3659 DEGs were assigned to 53 level-2 GO terms [22] according to three gene ontology classes, including 25 for biological process, 14 for cellular components, and 14 for molecular function (Fig. 2). For the cellular component group, the most represented category was cell part, cell and organelle. For biological process, metabolic process and cellular process were highly represented. Regarding molecular function, the category of catalytic activity was the most represented GO term, followed second by the category of binding.

### Functional classification by COG

**Fig. 3:**
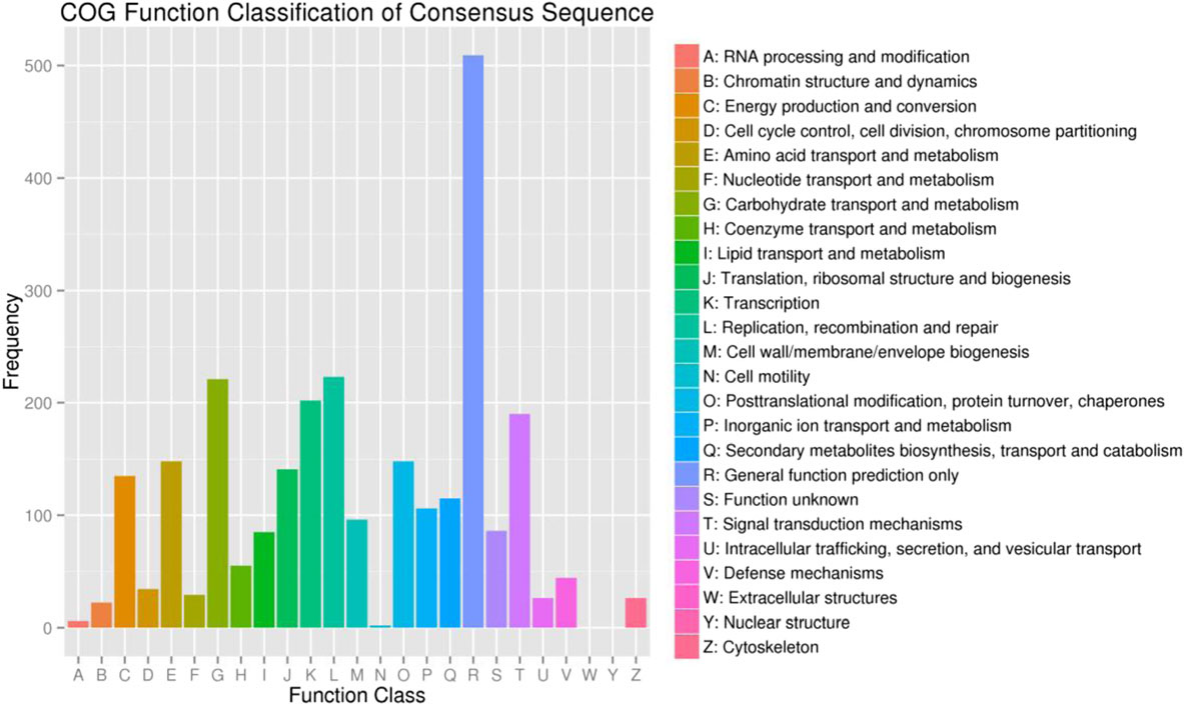
Clusters of Orthologous Groups (COG) annotations of *S. salsa* dimorphic seed unigenes are shown. A total of 1900 COG functional annotations were obtained, which were classified into 25 functional categories. The Y-axis indicates the number of unigenes in a specific functional cluster

COG classification, which is a tool for the genome-scale analysis of protein function and evolution, was carried out to assign functional information to DEGs [20]. Among the 25 COG categories, the cluster for “general function prediction only” had the largest number, followed by “replication, recombination and repair”, “carbohydrate transport and metabolism”, “transcription”, and “signal transduction mechanisms” (Fig. 3). There were no genes assigned in the “nuclear structure” and “intracellular trafficking, secretion, and vesicular transport” (Fig. 3).

### Functional classification by KEGG

A total of 1102 DEGs were assigned to 111 KEGG pathways. Most of these DEGs were mapped to “oxidative phosphorylation”, “ribosome”, “glycolysis/gluconeogenesis”, “starch and sucrose metabolism”, “amino sugar and nucleotide sugar metabolism”, “protein processing in endoplasmic reticulum”, and “plant hormone signal transduction” (Additional file 2: Table S1), which indicate that hormone content and carbohydrate metabolism may lead to different germination characteristics between brown and black seeds.

### Genes involved in fatty acid and embryo development

**Fig. 4:**
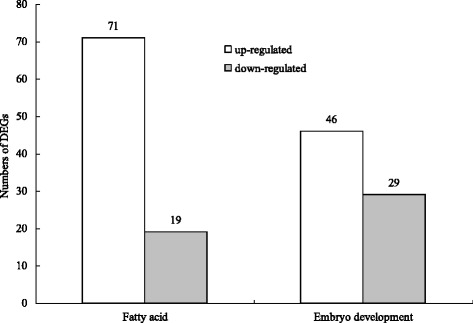
The number of DEGs related to fatty acid and embryo development of *S. salsa* dimorphic seeds

**Fig. 5:**
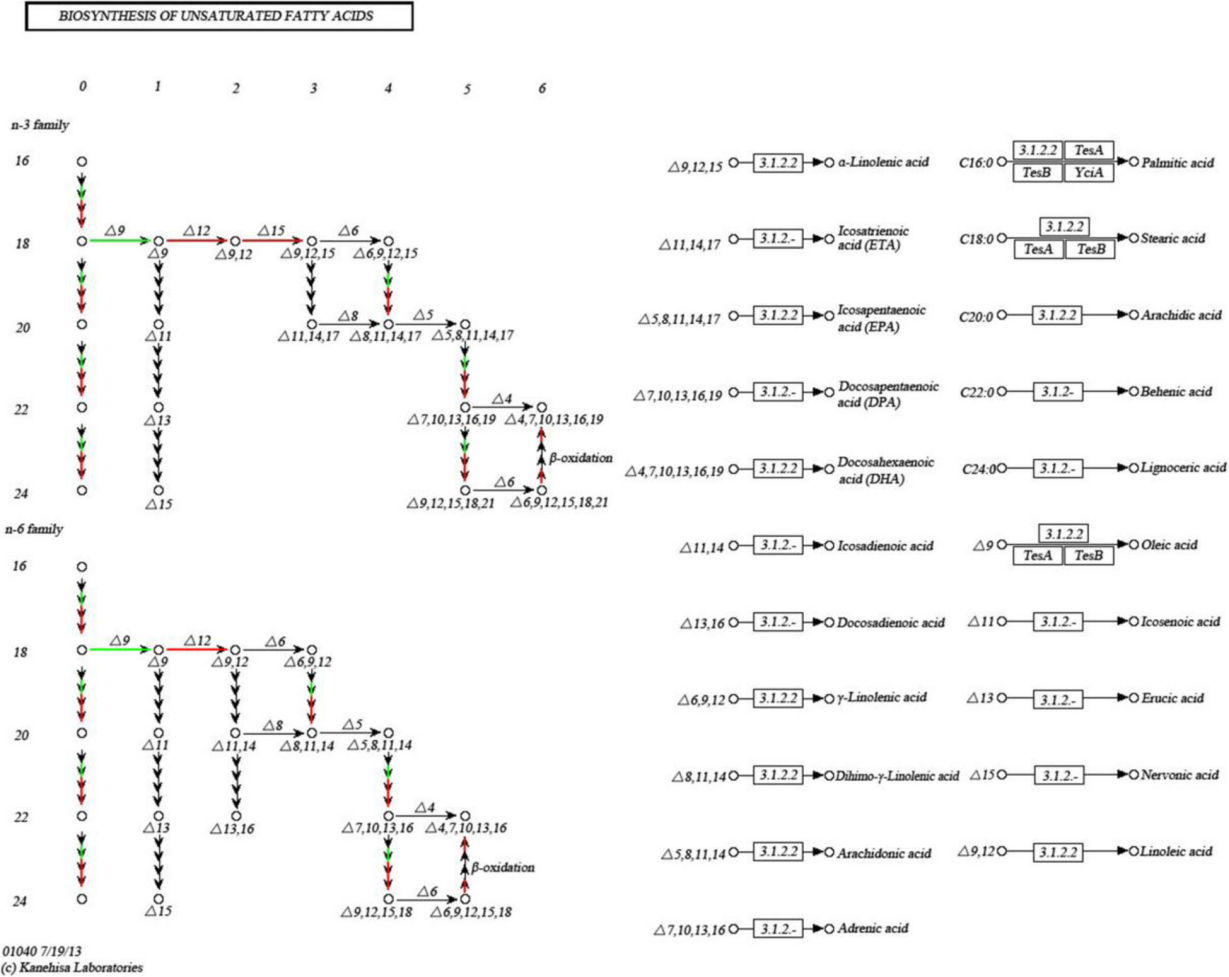
KEGG map of the unsaturated fatty acid biosynthesis pathway. It was an analysis of DEGs that compared brown seeds samples with black seeds. Detailed gene and expression data are provided in Additional file 5: Table S4. Red indicates the corresponding DEGs that were upregulated in brown seeds, green indicates the corresponding DEGs that were downregulated in brown seeds, blue indicates some of the corresponding DEGs that were downregulated and the others that were upregulated, and those without any color indicate the expression level of corresponding genes that did not change

Through transcriptome sequencing, 75 genes may be related to embryo development (Additional file 3: Table S2) and 90 genes related to the synthesis and metabolism of fatty acid were also obtained (Additional file 4: Table S3). Most of these genes, which were involved in fatty acid and embryo development, were also upregulated in brown seeds, compared to black seeds (Fig. 4). A total of 10 DEGs were annotated to “biosynthesis of unsaturated fatty acids” by KEGG analysis (Additional file 5: Table S4, Fig. 5). For the biosynthesis of unsaturated fatty acids, genes encoding 3-oxoacyl-[acyl-carrier protein] reductase, acyl-[acyl-carrier-protein] desaturase and stearoyl-CoA desaturase (Delta-9 desaturase) were downregulated. Genes related to very-long-chain enoyl-CoA reductase, acyl-lipid omega-3 desaturase (Delta-15 desaturase), acyl-lipid omega-6 desaturase (Delta-12 desaturase), and *ACAA1* (acetyl-CoA acyltransferase 1) were upregulated (Additional file 5: Table S4, Fig. 5).

### Genes involved in betaine, proline, sodium, chloride, potassium and calcium accumulation

**Fig. 6:**
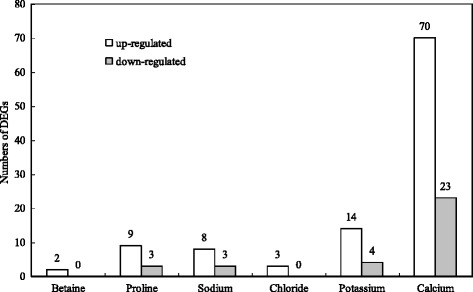
The number of DEGs related to betaine, proline, sodium, chloride, potassium, calcium of *S. salsa* dimorphic seeds

Numerous DEGs were annotated to the GO classification, and may be related to betaine, proline, sodium, chloride, potassium, and calcium accumulation, which were 2, 12, 11, 3, 18, and 93, respectively (Fig. 6). Most DEGs such as genes related to sodium ion transport (GO: 0006814), chloride transport (GO: 0006821), voltage-gated chloride channel activity (GO: 0005247), potassium ion transmembrane transport (GO: 0071805), cellular potassium ion homeostasis (GO: 0030007), potassium ion transmembrane transporter activity (GO: 0015079), and inward rectifier potassium channel activity (GO: 0005242) were upregulated in brown seeds, compared to black seeds (Additional file 6: Table S5).

### Genes involved in auxin (IAA), abscisic acid (ABA), cytokinins (CTK), salicylic acid (SA), jasmonic acid (JA), brassinosteroid (BR), ethylene (ETH) and gibberellins (GAs)

**Fig. 7:**
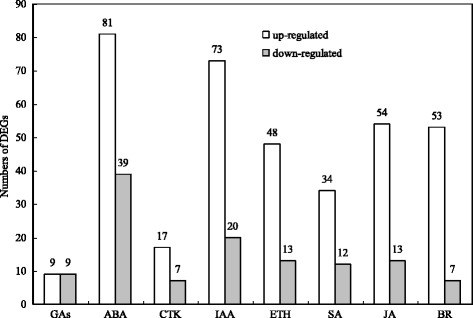
The number of DEGs related to plant hormones of *S. salsa* dimorphic seeds

**Fig. 8:**
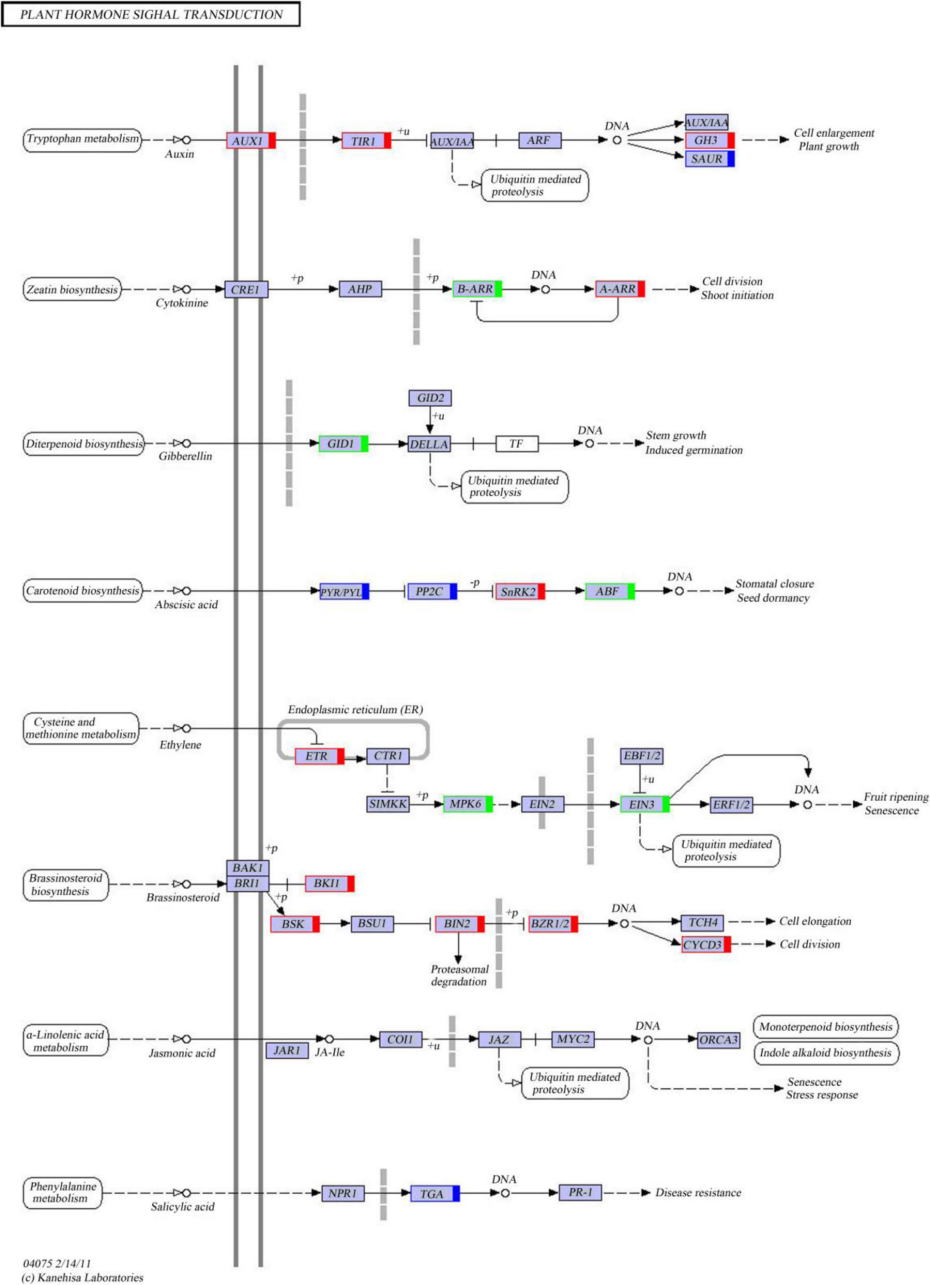
KEGG map of the plant hormone signal transduction pathway. This is an analysis of DEGs, comparing the samples of brown seeds with black seeds. Detailed gene and expression data was provided in Additional file 8: Table S7. Red indicates the corresponding DEGs that were upregulated in brown seeds, green indicates the corresponding DEGs that were downregulated in brown seeds, blue indicates some of the corresponding DEGs that were downregulated and the others that were upregulated, and those without any color indicate the expression level of corresponding genes that did not change

According to the GO annotations, 367 genes were found, which may be related to endogenous hormones. These genes may participate in the hormone biosynthetic process, metabolic or process signaling pathway, and polar transport from all DEGs (Additional file 7: Table S6). Among these, 283 genes were upregulated and 84 genes were downregulated in brown seeds, compared to black seeds (Fig. 7). Most of these genes were related to abscisic acid and auxin (Fig. 7). For example, 38 genes were annotated in “plant hormone signal transduction” by KEGG analysis (Fig. 8, Additional file 8: Table S7), and 11 genes were annotated to the auxin signal transduction process, in which most of genes were upregulated (the proportion was 81.82%). These genes were auxin influx carrier (*AUX1*), transport inhibitor response-1 (*TIR1*), auxin responsive GH3 gene family (*GH3*), and SAUR family protein (*SAUR*). Furthermore, *AUX1*, *TIR1* and *GH3* were upregulated. There were five genes (three genes were upregulated and two genes were downregulated) encoding SAUR. In the cytokinin signal transduction process, *A-ARR* (two-component response regulator ARR-A family: ARR4 and ARR9) was upregulated, and *B-ARR* (two-component response regulator ARR-B family: ARR11) was downregulated. In the gibberellin signal transduction process, *GID1* (gibberellin-insensitive dwarf 1: GID1C) was downregulated. In the abscisic acid signal transduction process, *SNRK2* (serine/threonine-protein kinase SRK2) was upregulated and *ABF* (ABA responsive element binding factor) was downregulated. There change to were three genes (two genes were upregulated and one gene was downregulated) encoding PYR/PYL (abscisic acid receptor PYR/PYL family), and two genes (one gene was upregulated and one gene was downregulated) encoding PP2C (protein phosphatase 2C). In the ethylene signal transduction process, ethylene receptor (*ETR*) was upregulated, while mitogen-activated protein kinase 6 (*MPK6*) and ethylene-insensitive protein 3 (*EIN3*) were downregulated. In the brassinosteroid signal transduction process, BRI1 kinase inhibitor 1 (*BKI1*), BR-signaling kinase (*BSK*), protein brassinosteroid insensitive 2 (*BIN2*), *BZR1/2* (brassinosteroid resistant 1/2: BZR1 and BZR2/BES1), and cyclin D3 (*CYCD3*) were upregulated. There are four genes (two genes were upregulated and two genes were downregulated) encoding TGA (transcription factor TGA) in the salicylic acid signal transduction process. There were no DEGs in the jasmonic acid signal transduction process (Fig. 8).

### Contents of endogenous hormones

**Fig. 9:**
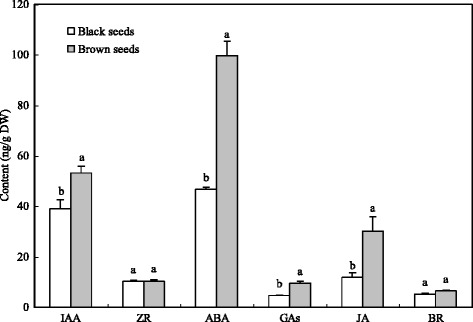
The concentrations of endogenous auxin (IAA), abscisic acid (ABA), cytokinins (ZR), jasmonic acid (JA), brassinosteroid (BR), and gibberellic acid (GAs) in *S. salsa* dimorphic seeds. Values are presented as mean + standard deviation (SD), *n* = 3. The difference in means with different letters was statistically significantly at *P* ≤ 0.05

GA, IAA, JA and ABA content were generally higher in brown seeds than in black seeds, especially for JA and ABA (Fig. 9). However, there was no significant difference in ZR and BR levels (Fig. 9).

### Concentration of Ca^2+^

**Fig. 10:**
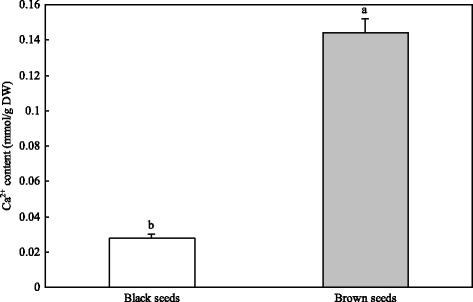
The concentration of Ca^2+^ in *S. salsa* dimorphic seeds. The values are presented as mean + standard deviation (SD), *n* = 3. The difference in means with different letters was statistically significant at *P* ≤ 0.05

The concentration of Ca^2+^ in brown seeds was much higher than that in black seeds; that is, the Ca^2+^ concentration in brown seeds was 5.3 times of that in black seeds (Fig. 10).

### qRT-PCR validation of RNA-Seq results

**Fig. 11:**
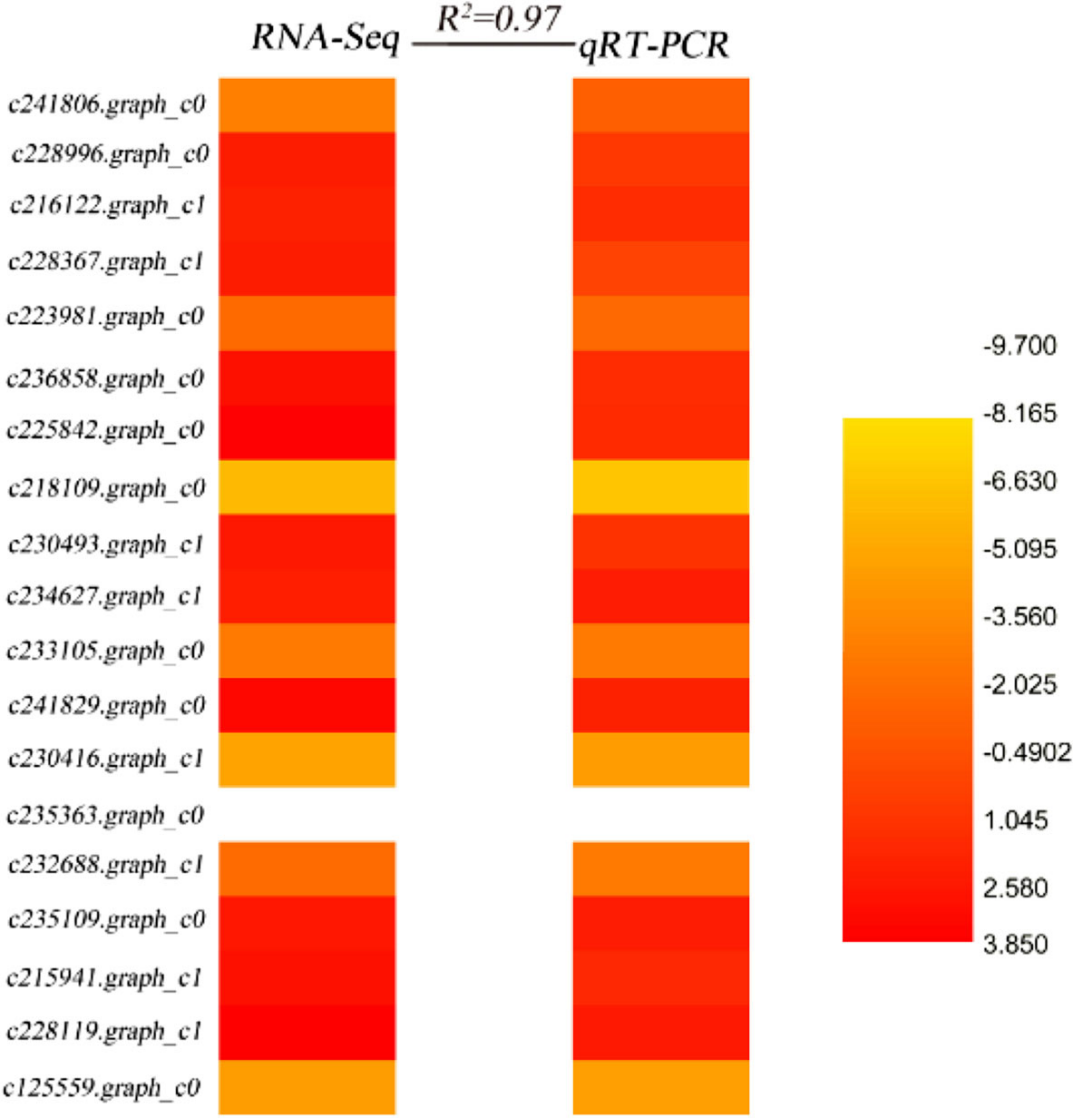
Validation of RNA-Seq results by qRT-PCR. The expression levels of 19 randomly selected genes used in this study were detected by RT-qPCR. R^2^ represents the correlation coefficient value between these two platforms. The numbers in the scale bar stand for log2FC values in RNA-seq and 2^− ΔΔCT^ in qRT-PCR, which were used to evaluate the correlation (R^2^). Primers are listed in Additional file 9: Table S8

Based on the results mentioned above, 20 pairs of primers were designed (Additional file 9: Table S8), and qRT-PCR was applied to validate the correlation against RNA-Seq. Results were basically identical with the sequencing results (*R* ^*2*^ = 0.97, Fig. 11), which indicates that the transcriptome sequencing results are reliable.

## Discussion

In the present study, a large number of differentially expressed genes were identified between brown and black seeds. Brown seeds of *S. salsa* were much heavier (bigger) and better developed than black seeds [9]. DEGs, according to the GO annotation of the transcriptome sequencing, may participate in the biological process of embryo development (the proportion of upregulated genes in brown seeds compared to black seeds was 61.33%); that is, they may play a positive role in embryo development. Seeds of *S. salsa* can produce oil, which contains several kinds of fatty acid with sufficient unsaturated fatty acids, especially linoleic acid [23]. In the biosynthesis of the unsaturated fatty acid signal transduction process, fatty acid desaturase, which almost exists in all organisms, performed a vital role [24]. Delta-9 desaturase can convert the saturated fatty acids into oleic acid by introducing the first double bond, and the delta-12 desaturase catalyze oleic acid into linoleic acid, which may further be converted by delta-15 desaturase into a-linolenic acid [25]. Genes that encode delta-12 desaturase had a relatively higher expression in brown seeds than in black seeds. It is possible that better developed embryos and more unsaturated fatty acids can provide more nutrition during rapid seed germination. This deduction needs to be further verified.

In a saline environment, seed germination was inhibited to a certain extent, and was affected by specific-ion toxicity or/and osmotic stress [26–28]. High concentration of inorganic ions such as Na^+^, K^+^ and Cl^−^ can lower water potential and help seeds absorb water from saline soils during germination [29, 30]. Liu et al. found that the concentration of Na^+^, K^+^, Cl^−^ and total ions of brown seeds was much higher than those of black seeds [31]. In addition, the concentration of Ca^2+^ revealed a similar tendency (Fig. 10). These present results of transcriptome sequencing indicate that most genes related to Na^+^, K^+^, Ca^2+^ and Cl^−^ accumulation were upregulated in brown seeds compared to black seeds (Fig. 6). Vacuolar cation/proton exchanger 3 (CAX3) is localized in the tonoplast, and is involved in ion vacuolar compartmentalization [32–34]. Punshon et al. found that the *CAX3* transcript was abundant in dry seeds of *Arabidopsis* [35]. *CAX3* is strongly expressed during seed germination [36, 37]. In the present study, *CAX3* was upregulated in brown seeds compared to black seeds of *S. salsa* (Additional file 6: Table S5). Therefore, *CAX3* may play an important role in nutrient acquisition in brown seeds. In addition, the high expression of *CAX3* may be contributed to the higher salt resistance of brown seeds compared to black seeds in *S. salsa*. Proteins of the CLC (chloride channel) family, which participate in Cl^−^ balance and prevent Cl^−^ toxicity under salt stress, has a potential physiology function in the salt tolerance of plants [38, 39]. In the present study, all genes related to chloride transport and voltage-gated chloride channel activity (CLC-c, CLC-f, and CLC-g) were upregulated in brown seeds, compared to black seeds of *S. salsa* (Additional file 6: Table S5). Genes that encode putative potassium transporter 12, two-pore potassium channel 1, and 1-aminocyclopropane-1-carboxylate are significantly upregulated, while the expression of genes that encode probable potassium transporter 13, potassium channel AKT1, and two-pore potassium channel 3 exhibited an opposite trend (Additional file 6: Table S5). The expression of genes may relate to Na^+^, K^+^, Ca^2+^ and Cl^−^ transport carriers or channels that can help regulate salt ions in and out, and reduce salt ion poison under salt stress [40]. It is possible that the interaction of these genes can maintain ion homeostasis in *S. salsa* dimorphic seeds during germination under salt stress.

In addition to the large number of inorganic ions, some organic osmotic regulation substances were also synthesized to lower the water potential of plants under salt stress [41–43]. Proline and betaine, which can adjust osmotic potential and stabilize biological macromolecules, are two important osmotic regulation substances [41, 44]. Compared to black seeds, genes that could encode betaine aldehyde dehydrogenase (*BADH*) were upregulated (Additional file 6: Table S5). Li et al. found that overexpressing the *BADH* gene, which was cloned from *Suaeda liaotungensis* in tobacco, could improve the salt tolerance of transgenic tobacco by producing betaine [45]. Therefore, the upregulation of *BADH* (Additional file 6: Table S5) may help brown seeds accumulate more betaine than that of black seeds. Yokoishi and Tanimoto found that an increase in betaine appeared to secure seed germination under salt stress in halophyte *Suaeda japonica* [46]. Genes (*fis1*) that may encode aldehyde dehydrogenase, which are most likely the P5CDH (delta-1-pyrroline-5-carboxylate dehydrogenase) enzyme related to the proline catabolic process to glutamate, were upregulated in brown seeds, compared to black seeds [47]. Poljakoff-Mayber et al. found that proline concentration was low in dry *Kosteletzkya virginica* seeds, and proline content increased when seeds were germinated under salt stress [48]. It is possible that proline keeps the developing embryonic axis in a resting state, and the synthesis of proline may be an adaptive strategy of the plant to prevent germination under stressful environments [49]. Therefore, the upregulation of *P5CDH* may not be related to the high ability of osmotic adjustment in brown seeds, but plays a protective role in brown seed germination under saline conditions.

Plant hormones have important effects on plant development with vanishingly low concentrations, as well as in seed dormancy and germination [50, 51]. Germination is crucial in the whole life cycle of plants, especially for halophytes in variable saline environments [12, 52]. Seed dormancy is a mechanism that can prohibit seed germination in unfavorable conditions [51]. There were large numbers of DEGs probably related to hormone (GAs, ABA, CTK, IAA, ETH, SA, JA, and BR) synthesis, transport and metabolism in the transcriptome sequencing of *S. salsa* dimorphic seeds (Additional file 7: Table S6). Except for genes for GAs, most genes may be related to other hormones in brown seeds were upregulated, compared to black seeds (Fig. 7). Correspondingly, the content of these hormones in brown seeds were higher than in black seeds, except for ZR and BR (Fig. 9). It is possible that the interactions between these hormones (hormone cross-talk) play profound effects on seed germination. For example, ABA can inhibit germination and induce dormancy, but GAs can stimulate germination [53]. Ethylene promotes seed germination possibly through the antagonism of ABA signaling [53]. JA and SA play a negative regulation of seed germination [54, 55]. It is possible that JA and ABA play a synergistic role during seed germination, but both synergism and antagonism can occur between the JA and ethylene pathways [56]. The auxin influence on seed germination is based on GAs, ABA and ETH regulations [57]. The signals of BR decrease the sensitivity of ABA, and promote seed germination [58]. In a controlled experiment, ZR and IAA content was much higher in brown seeds than in black seeds of *S. salsa*, which indicate that ZR and IAA may take important functions as growth regulators, and may promote the development of brown seeds, compared to black seeds [59]. CTK and IAA can also increase the salt tolerance of seeds in certain plant species [60]. Therefore, those upregulated genes related to CTK and IAA may promote the development of brown seeds. As a result, brown seeds are much bigger and have a higher ability of salt tolerance than black seeds [9]. Seed germination/dormancy appears to be controlled by the GA/ABA ratio rather than by absolute hormone levels [22, 61]. Most of the genes involved in the gibberellins biosynthetic process (GO: 0009686) were upregulated (Additional file 7: Table S6). Genes that encode GA 20-oxidase (c241829.graph_c0, c223973.graph_c1) (Additional file 7: Table S6), which is a key enzyme that catalyses the last three steps related to GA biosynthesis, were also highly upregulated [62]. It is possible that the upregulation of these genes positively accumulates GAs and further affects the germination/dormancy of brown seeds. ABFs, which belong to a distinct subfamily of bZIP proteins, are induced by ABA and various stress treatments [63]. SnRK2s can phosphorylate ABFs to activate ABA-responsive gene expression [64]. In these present results, *ABF* was downregulated in brown seeds compared to black seeds (Fig. 8). Therefore, *ABFs* may increase the degree of dormancy in black seeds of *S. salsa*. The vital role of ethylene is performed in the dormancy release and germination to non-dormant seeds [65]. ETR is a ethylene receptor that negatively regulates ethylene signaling [66]. The germination of ethylene-insensitive mutant (*etr* mutant) was inhibited at the same conditions compared with wild-type seeds of *Arabidopsis* [67, 68]. These present results indicate that the upregulation of *ETR* in brown seeds (Fig. 8) is probably one of reasons for the quick and high germination of brown seeds. BR is considered to have performed a vital role in antagonizing seed dormancy and stimulating germination [58]. In the present study, many genes related to BR were upregulated in brown seeds, compared with black seeds (Fig. 7, Additional file 7: Table S6), which indicate that BR may also play a role in the quick germination of brown seeds in *S. salsa*.

## Conclusions

In conclusion, the present study reported a transcriptome view of the complexity of gene expression in matured dimorphic seeds of *S. salsa*. This can help to further investigate the adaptation of seed dimorphic plants to variable saline environments. Further genetic and biochemical analysis should be performed to detect the functions of key genes related to embryo development, inorganic and organic substance accumulation, dormancy and germination in *S. salsa* dimorphic seeds.

## Methods

### Plant material and RNA extraction

Approximately 50 plants of *Suaeda salsa* L. from saline inland (N37°20′, E118°36′) were randomly collected in the Yellow River Delta in Shandong province, China, when the seeds became completely mature. After seeds were air-dried, the brown and black seeds were separated; and the seeds were immediately frozen in liquid nitrogen and stored at −80 °C. Total RNA was extracted from pooled samples of brown or black seeds using a total RNA kit (E.Z.N.A.TM Plant RNA Kit R6827-01; OMEGA, Beijing, China), according to manufacturer’s instructions. Then, these were examined using a NanoDrop ND1000 spectrophotometer (NanoDrop Technologies, Wilmington, DE, USA) and characterized on 1% agarose gel electrophoresis. The standards applied were OD260/OD230 ≥ 1.0, and 1.8 ≤ OD260/OD280 ≤ 2.2. The RNA integrity number (RIN) values (>8.0) of these samples were assessed using an Agilent 2100 Bioanalyzer (Santa Clara, CA, USA). At least 20 μg of RNA was pooled in an equimolar fashion from each of the samples. Two replicates of black seeds (T1 and T2) or brown seeds (T3 and T4) were performed in this study, and each sample had 0.5 g of seeds.

### Illumina sequencing and de novo assembly

In this experiment, mRNA was enriched and purified with oligo (dT)-rich magnetic beads, and the cDNA libraries were constructed. We tested the library insert fragment size in 1.8% agarose gel electrophoresis. Then, QPCR was performed to enrich the purified cDNA template. Finally, the libraries were sequenced using Illumina HiSeq™2500 with a paired-end strategy at the Illumina Sequencing Services of Biomarker Technologies Co, LTD. (Beijing, China). The original reads obtained from the sequencing data was evaluated and filtered. The Trinity software (http://trinityrnaseq.sourceforge.net/) was adopted to assemble the high quality data after these were filtered, which did not include the Gap in the final length of the constructed transcription. Then, non-redundant unigenes were generated by de novo assembly.

### Gene expression analysis

RPKM (Reads per Kilobase per Million mapped reads) values [69] were used to estimate all unigenes of the gene expression in the brown and black seeds of *S. salsa*. DEGs in the brown and black seeds of *S. salsa* were filtered using the DESeq software [70]. Differentially expressed genes were selected on the basis of a threshold of false discovery rate (FDR) <0.01 and an absolute value of log2 Fold Change (log2FC) >1.

$$ \mathrm{RPKM}=\mathrm{total}\  \mathrm{exon}\  \mathrm{reads}/\left(\mathrm{mapped}\  \mathrm{reads}\left(\mathrm{millions}\right)\times \mathrm{exon}\  \mathrm{length}\ \left(\mathrm{kb}\right)\right) $$

### Functional annotation

The unigene sequences were compared with the Nr [17], Swiss-Prot [19], GO [21], COG [20], and KEGG [18] databases to identify and annotate the obtained unigenes using Blast [71] software.

### Determination of hormone contents

The contents of endogenous IAA, ABA, ZR, JA, BR and GAs in black and brown seeds were analyzed by enzyme linked immunosorbent assay (ELISA) at the College of Crop Sciences, China Agricultural University [59, 72, 73]. Three replicates were used for brown and black seeds.

### Measurement of Ca^2+^ concentration

The sample (0.3 g of dry black or brown seeds for each sample) was prepared by microwave digestion in HNO_3_-H_2_O_2_, and ultrapure water was added to obtain a final volume of 25 mL [74]. Then, these were analyzed using an inductively coupled plasma (ICP-OES) spectrometer (Optima 7300 DV, Perkin-Elmer, USA). Three replicates were used for brown and black seeds.

### qRT-PCR analysis

In order to test the expression of the target gene transcript, a total of 19 DEGs were randomly selected for real-time PCR analysis. All primers were designed using the Beacon Designer software (version 7.9). The housekeeping gene actin (GenBank ID: EU429457) was used as an internal standard [75]. Real-time PCR was carried out in a fluorometric thermal cycler (Bio-Rad CFX96TM Real-time PCR System, California, USA) using SYBR Premix Ex TaqTM (TaKaRa) in 20-μL reaction mixtures containing 10 μL of 2 × SYBR Premix Ex TaqTM (TaKaRa), 50 ng of cDNA and 0.8 μM of primer pairs (actin and target gene). The relative expression of each gene was calculated by 2^− ΔΔCT^ method [76]. Three replicates were performed.

### Statistical analysis

Data for concentrations of endogenous hormones and Ca^2+^ were analyzed by one-way ANOVA. SAS™ software [77] was used for statistical analysis.

## Additional files

Additional file 1: Figure S1. Length distribution of unigenes identified in the transcriptome of *S. salsa* dimorphic seeds. (DOC 141 kb)

Additional file 2: Table S1. Functional classification by KEGG pathways in *S. salsa* dimorphic seeds. (XLS 20 kb)

Additional file 3: Table S2. DEGs related to embryo development according to GO function annotation. (XLS 27 kb)

Additional file 4: Table S3. DEGs related to fatty acid according to GO function annotation. (XLS 31 kb)

Additional file 5: Table S4. DEGs in the biosynthesis of the unsaturated fatty acid pathway. (XLS 10 kb)

Additional file 6: Table S5. DEGs related to osmotic regulation substances according to GO function annotation. (XLS 57 kb)

Additional file 7: Table S6. DEGs related to plant hormones according to GO function annotation. (XLS 118 kb)

Additional file 8: Table S7. DEGs in the plant hormone signal transduction pathway. (XLS 23 kb)

Additional file 9: Table S8. Primer pairs for real-time quantitative PCR. (XLS 17 kb)

## Abbreviations

A-ARR: Two-component response regulator ARR-A family
ABA: Abscisic acid
ABF: ABA responsive element binding factor
ACAA1: Acetyl-CoA acyltransferase 1
AUX1: Auxin influx carrier
BADH: Betaine aldehyde dehydrogenase
B-ARR: Two-component response regulator ARR-B family
BIN2: Protein brassinosteroid insensitive 2
BKI1: BRI1 kinase inhibitor 1
BR: Brassinosteroid
BSK: BR-signaling kinase
BZR1/2: Brassinosteroid resistant 1/2: BZR1 and BZR2/BES1
CAX: Vacuolar cation/proton exchanger 3
CLC: Chloride channel
COG: Clusters of orthologous groups
CTK: Cytokinins
CYCD3: Cyclin D3
DEGs: Differentially expressed genes
EIN3: Ethylene-insensitive protein 3
ETH: Ethylene
ETR: Ethylene receptor
FDR: False discovery rate
GAs: Gibberellins
GH3: Auxin responsive GH3 gene family
GID1: Gibberellin-insensitive dwarf 1
GO: Gene ontology
IAA: Auxin
JA: Jasmonic acid
KEGG: Kyoto encyclopedia of genes and genomes
MPK6: Mitogen-activated protein kinase 6
Nr: NCBI non-redundant
P5CDH: Delta-1-pyrroline-5-carboxylate dehydrogenase
PP2C: Protein phosphatase 2C
PYR/PYL: Abscisic acid receptor PYR/PYL family
RIN: RNA integrity number
RNA-seq: RNA-sequencing
RPKM: Reads per KB per million
SA: Salicylic acid
SAUR: SAUR family protein
SNRK2: Serine/threonine-protein kinase SRK2
TGA: Transcription factor TGA
TIR1: Transport inhibitor response 1
